# Combining genomic selection with genome-wide association analysis identified a large-effect QTL and improved selection for red rot resistance in sugarcane

**DOI:** 10.3389/fpls.2022.1021182

**Published:** 2022-10-31

**Authors:** Anthony O’Connell, Jasmin Deo, Emily Deomano, Xianming Wei, Phillip Jackson, Karen S. Aitken, Ramaswamy Manimekalai, Krishnasamy Mohanraj, Govinda Hemaprabha, Bakshi Ram, Rasappa Viswanathan, Prakash Lakshmanan

**Affiliations:** ^1^ Sugar Research Australia Limited, Brisbane, QLD, Australia; ^2^ Commonwealth Scientific and Industrial Research Organisation (CSIRO) Agriculture and Food, Queensland Bioscience Precinct, St Lucia, QLD, Australia; ^3^ Sugarcane Breeding Institute, Coimbatore, India; ^4^ Sugarcane Research Institute, Guangxi Academy of Agricultural Sciences, Nanning, China; ^5^ Interdisciplinary Research Center for Agriculture Green Development in Yangtze River Basin (CAGD), College of Resources and Environment, Southwest University, Chongqing, China; ^6^ Queensland Alliance for Agriculture and Food Innovation, University of Queensland, Brisbane, QLD, Australia

**Keywords:** sugarcane, GWAS, genome wide association study, red rot, molecular breeding, genomic selection

## Abstract

Red rot caused by the fungus *Colletotrichum falcatum* is the main disease limiting sugarcane productivity in several countries including the major producer India. The genetic basis for red rot resistance is unclear. We studied a panel of 305 sugarcane clones from the Australian breeding program for disease response phenotype and genotype using an Affymetrix^®^ Axiom^®^ array, to better understand the genetic basis of red rot resistance. SNP markers highly significantly associated with red rot response (≤ 10^-8^) were identified. Markers with largest effect were located in a single 14.6 Mb genomic region of sorghum (the closest diploid relative of sugarcane with a sequenced genome) suggesting the presence of a major-effect QTL. By genomic selection, the estimated selection accuracy was ~0.42 for red rot resistance. This was increased to ~0.5 with the addition of 29 highly significant SNPs as fixed effects. Analysis of genes nearby the markers linked to the QTL revealed many biotic stress responsive genes within this QTL, with the most significant SNP co-locating with a cluster of four chitinase A genes. The SNP markers identified here could be used to predict red rot resistance with high accuracy at any stage in the sugarcane breeding program.

## Introduction

Cultivated sugarcane (*Saccharum* spp. inter-specific hybrids) is a major food and industrial crop grown in more than 110 countries in the tropics and sub-tropics (FAO, 2020). Globally, it is the fifth most valuable crop economically, providing >80% of the sugar and ~35% of the bioethanol in the world. Brazil and India combined account for more than 50% of sugarcane production in the world. Like other crops, maintaining resistance to important diseases is a major objective of sugarcane breeding programs worldwide (Heinz, 1987; Jackson, 2018). Sugarcane red rot disease caused by the fungus *Colletotrichum falcatum* Went has been reported in 77 countries and is the most damaging sugarcane disease in India, Pakistan, Thailand, Nepal, Myanmar and Vietnam (Viswanathan et al., 2018). The disease causes rotting of sugarcane stalk tissue, affecting cane yield and sugar quality. Inversion of stored sucrose by the pathogen affects sugar juice quality, causing reduced sugar recovery in sugar mills. In India, large-scale red rot epidemics have occurred every decade since its first appearance in 1901, resulting in large economic loss and the removal of highly productive and widely cultivated varieties from production (Viswanathan et al., 2018). By contrast in some other countries including Australia, red rot disease is present but is observed rarely and has only a very small impact on commercial cane production. Resolving the underlying reasons for the differing impact between countries is of interest and potential practical importance.

Selection and deployment of resistant varieties is the most common strategy used to manage red rot in affected sugarcane industries (Viswanathan et al., 2018). In India no variety is released for commercial production unless it has resistance to red rot. However, breakdown of red rot resistance is common (Viswanathan, 2021). In India, Co 205, the first hybrid sugarcane cultivar (i.e. first cultivar with a *Saccharum spontaneum* ancestor) that was released in 1918, and which quickly became dominant in northern India, succumbed to red rot within a few years after release (Chona, 1980). Over the next 100 years, nearly all sugarcane cultivars in India, which were resistant at the time of commercial release, became susceptible and succumbed to the disease within a period of 3 to 20 years following release (Chona, 1980; Viswanathan, 2021). Unlike other sugarcane pathogens, new *C. falcatum* pathotypes with varying degrees of virulence are frequently formed through mutations and parasexual recombination, causing resistance breakdown (Viswanathan et al., 2020). Gain and loss of virulence and occasional emergence of super-virulent pathotypes have been reported (Viswanathan et al., 2020). Although the underlying mechanism for host-resistance breakdown is unclear, development of new *C. falcatum* pathotypes and their adaptation to new varieties contributes to resistance breakdowns and disease epidemics (Viswanathan et al., 2021).

Studies on inheritance of red rot resistance have reported a moderate to high narrow-sense heritability and high broad sense heritability (Babu et al., 2010; Alarmelu et al., 2010). This indicates that both additive genetic variance (i.e. variation due to presence or absence of alleles) and non-additive (i.e. dominance variation due to combinations of alleles at particular loci, or epistasis variation due to interactions between alleles at different loci) are important. The high values (>0.90) reported for broad-sense also indicates potentially stronger genetic control of response to the disease compared with environmental factors (Ram et al., 2006). A major source of resistance in sugarcane cultivars is believed to be derived from *S. spontaneum* ancestors (Natarajan et al., 2001). It is also believed that a combination of vertical resistance (due to race specific large gene effects) and horizontal resistance (non-race specific resistance) contributes to overall resistance to red rot (Alarmelu et al., 2010; Babu et al., 2010). An association mapping study on red rot resistance in sugarcane by Singh et al. (2016) identified several markers explaining between 10-17% of variation in resistance scores which was independent of population structure. However, as noted by the authors, this study was limited in statistical power to some extent by the relatively small size of the association mapping panel used (116 clones) and because the majority of genotypes screened fell into the single category of being moderately resistant.

Determining the genetic basis of resistance to the disease through association mapping, and whether it is the same or different in other affected countries, could allow breeders to more effectively select for durable resistance. In Australia, despite red rot not currently being a serious disease, it is of interest to sugarcane breeders in Australia and India to better understand the genetic basis of resistance for two reasons. Firstly, this information may be used in future marker assisted breeding programs to eliminate susceptibility in parental or progeny populations. Secondly, this information may be coupled with future studies to determine the likely reaction of Australian germplasm to races of *C. falcatum* in India. If QTL identified as conferring resistance in Australian populations are not present in Indian breeding programs, these may provide a useful target for introduction by the latter. Conversely, if these QTLs are already present in clones susceptible to red rot in India, this would indicate a likely biosecurity vulnerability to guard against or address.

Genetic studies in sugarcane are usually more challenging in comparison to those with similar goals in other major crops. This is at least partly due to the large and complex genome of sugarcane, which is highly heterozygous and polyploid (frequently aneuploid). Genome wide association studies (GWAS) have been conducted in sugarcane research to identify specific QTL and associated DNA markers for a range of traits including fibre composition (Yang et al., 2019), yield traits (Gouy et al., 2015; Racedo et al., 2016; Barreto et al., 2019; Yang et al., 2020), yellow leaf virus resistance (Debibakas et al., 2014; Pimenta et al., 2021), leaf angle (Chen et al., 2022) and red rot (Singh et al., 2016) using a combination of diversity array technology (DArT), simple sequence repeats (SSR) amplified fragment length polymorphism (AFLP) and SNP markers in populations of 100-300 sugarcane genotypes (clones).

GWAS studies aim to identify individual markers correlated with traits of interest, but are constrained by limited statistical power to identifying only QTL with moderate to large effect, particularly when population sizes are small (eg. panels with<1000 genotypes). This is a potentially important limitation because it is clear that most commercially important traits in sugarcane are controlled by relatively small effects of large numbers of genes. Resolution of marker-trait association by some past studies may also be limited by the small number of available markers relative to the large size of the sugarcane genome.

More recently genomic selection (GS) has been advocated as a potentially useful approach that may be used in sugarcane breeding programs to more accurately select for traits controlled by large numbers of small effect QTL. In sugarcane this method has been applied with encouraging results (Gouy et al., 2013; Deomano et al., 2020; Yadav et al., 2020; Hayes et al., 2021; Islam et al., 2021). Where individual QTL of large effect are identified, GS can also be used in models with single QTL effects identified using genome wide association studies (GWAS) to maximise prediction accuracy (Bernardo, 2014). The development of a high density sugarcane Affymetrix^®^ Axiom^®^ array containing over 58K single nucleotide polymorphism (SNP) (Aitken et al., 2017) allows for low-cost screening of sugarcane germplasm with a far larger number of markers providing greater genome coverage and marker density. This improves the likelihood of identifying markers in close proximity to the gene or QTL underlying a trait of interest.

Here we screened a population of 305 clones representative of clones generated routinely in the Sugar Research Australia (SRA) sugarcane breeding program for response to red rot disease. These same clones were also genotyped using the Affymetrix^®^ Axiom^®^ SNP array to identify specific markers linked to resistance to this disease. We examined the results from analysing data using GWAS and GS for resistance to red rot. We evaluated the accuracy of GS using pedigree, markers and a combination of both to predict red rot resistance. The population of clones studied were also characterised for tonnes of cane per hectare (TCH) and commercial cane sugar (CCS) content, and results from these traits provided a comparison for red rot. In particular, GS prediction accuracies for red rot resistance were compared with TCH and CCS which have been studied previously (Deomano et al., 2020; Hayes et al., 2021). We also determined the genomic location of SNP markers that were strongly associated with red rot resistance in order to help identify candidate genes that may be related causally to the response to red rot. Highly significantly associated SNPs were located in close proximity to multiple stress responsive genes which have previously been identified in transcription studies and this information may help resolve the genetic control of red rot resistance in sugarcane, and lead to more robust markers for clone selection in the future.

## Materials and methods

### Genetic materials, field experiment and yield measurements

Three hundred and five clones from a final stage regional selection trial of the SRA sugarcane were used for the study. These clones were representative of those routinely generated and evaluated in the Australian commercial sugarcane breeding program, apart from three clones which were commercial standard cultivars. The clones were derived from 186 different parent clones and 166 crosses, also representative of those used and generated in the Australian sugarcane breeding program.

A field trial was established at Kalamia mill estate in north Queensland, Australia to measure cane yield (tonnes/ha, TCH) and commercial cane sugar (% fresh weight, CCS) (BSES, 1984). About ten percent (30) of the 305 clones evaluated were replicated twice to measure error variance (see section 2.5), while other clones were planted in one replicate. Each individual plot had four rows, 10 m in length, and there was an interrow spacing of 1.6 m. The trial was planted at Kalamia on 6 May 2013, and then cultivated and harvested following recommended local commercial crop management practices. Cane yield (TCH) and CCS were determined at harvest (12-month-old crop) following standard methods used in the SRA breeding program (BSES, 1984), and data for these two traits from the plant crop (first year) and ratoon crop (second year) was collected and analysed.

### Isolation and culturing of *Colletotrichum falcatum*

Sugarcane stalks showing typical red rot symptoms were sourced from a sugarcane farm near Mackay, Queensland, Australia. Stem cuttings were thoroughly cleaned with water, sprayed with 70% ethanol and split longitudinally with a sterile knife in a laminar-flow hood. Small pieces of infected tissues were isolated under sterile condition and cultured on potato dextrose agar in Petri dishes and stored at room temperature in dark conditions for several days. Fungal colonies with typical *C. falcatum* morphology were sub-cultured regularly to produce pure isolates. Pathogen identity of isolates was confirmed by conidia, culture morphology, and red rot symptoms in sugarcane. Sporulating fungi were suspended in deionised water and mixed with a kitchen blender to produce inoculum for red rot screening trials. Conidial concentration of the inoculum was adjusted to 1 million conidia per ml.

### Screening of breeding trial clones for red rot resistance

The level of resistance to red rot was observed for all 305 clones in the trial following methods developed by the Sugarcane Breeding Institute, Coimbatore, India and described by Mohanraj et al. (1997). These procedures were briefly as follows. Six-month-old cane stalks with an intact shoot top were used for the screening trial. After stripping off the older leaves and trimming the remaining ones to half-length, the upper part of the stalk with seven visible nodes was cut and inoculated by wrapping cotton swabs moistened with 5 mL of *C. falcatum* conidial suspension or water (control) around the second and third visible nodes from the top. Inoculated stalks were positioned upright with cut end inside wet sand and maintained in a growth chamber set at 30°C, > 90% humidity and constant light for two weeks. The experiment design was a randomised complete block with four replicated stalks inoculated for each clone. A highly resistant standard (negative control) and a highly susceptible standard (positive control) were also included in this experiment.

After two weeks the stalks were split longitudinally and disease symptoms were scored following the metrics described previously (Srinivasan and Bhat, 1961): shoot top condition (0 = healthy, 1 = dry/yellow), lesion width above inoculated node (0, 1, 2, or 3), nodal transgression of lesion (0, 1, 2, or 3 nodes transgressed) and occurrence of white spots (0, 1 = restricted, 2 = progressive) to give a total score from 0 (highly resistant) to 9 (highly susceptible).

### Genotyping and SNP marker screening

All clones were genotyped using an Affymetrix Axiom SNP array developed for sugarcane with 58,028 SNPs, previously screened and chosen based on quality parameters and polymorphism in Australian and Brazilian parental clones (Aitken et al., 2017). High-quality DNA was extracted from leaf tissues using a standard CTAB method, treated with proteinase K and purified on a Qiagen column. The Axiom assay was performed on 96-sample Axiom array following the procedure described by Affymetrix (http://media.affymetrix.com/support/downloads/manuals/axiom_2_assay_auto_workflow_user_guide.pdf ). DNA samples that had a dish quality control (DQC) measure of less than 0.82 or a quality control (QC) call rate of less than 97% were excluded from the analysis. Allele calling was performed using generated CEL files with Axiom Analysis Suite (1.1.0.616) (http://media.affymetrix.com/support/downloads/manuals/axiom_genotyping_solution_analysis_guide.pdf). For each polymorphic marker, all genotypes were given a marker score of 1 if only the most frequent allele was present (i.e. homozygous for this allele), 0 if both alleles were present (i.e. heterozygous), and − 1 if only the minor allele was present. Markers in which one of these three classes occurred for > 98% of the clones were deleted. A total of 56, 788 polymorphic markers were retained for further analysis after the above filtering. For each marker, missing values were replaced with the most frequent allele within a marker.

### Analysis of phenotypic data

TCH, CCS, red rot rating were analysed under a mixed model framework using a commercial R package, Asreml-R.

For TCH and CCS, an optimal linear mixed model was first determined for each trial crop class data from fitting different fixed, random and residual effects (Butler et al., 2017). For fixed effects replicate, linear row and linear column were considered. Clone is fitted as a random factor as well as spline row and spline column. Spatial variation along the row and along the column was also accounted for. The best model for each trial crop data were then used to fit a model to the multiple trial crop class data. Trial crop is added as a fixed effect in the model. Also, a correlated genetic variance was fitted to the G matrix to account for genotypic variance heterogeneity and correlated measurements between trial crop classes. On the other hand, an unstructured general correlation model and heterogenous variance form was used for the R matrix to account for heterogenous and correlated error variances of trial crop classes.

For red rot, the linear mixed model can be described as follows:


y = μ + date + rep/date + clone + e

where *y* is the measurement of total score from each plot, *μ* is a grand mean and *e* is the residual effects. Date, replicates within date (rep/date) and clone were set as random effects. All the random effects were assumed to follow iid N(0, Iσ^2^). Similarly, BLUPs of clonal effects and the broad-sense heritability were estimated from the model, which were used as observed values in genomic selection.

### Genome wide association studies

The association between each individual SNP marker and Red Rot resistance, TCH, and CCS was analysed using ASReml-R package based on the following mixed model:


y = a + d + r/d + SNP + u + e

where *y* is BLUP of a trait obtained from the above analysis, *a* i the intercept, *SNP* is a fixed effect of SNP, *u* is a polygenic effect ~ N(0, Aσ^2^α) where A is the numerator relationship matrix and σ^2^α is the polygenic variance; *e* = random residual effects ~ iid N(0, $I_{d}⊗\sigma \frac{2}{e_{d}}$)

Linkage disequilibrium between highly significant SNPs (P<10^-7^) was calculated using Haploview software (Barrett et al., 2005). SNPs with an LD score of ≥ 0.8 were allocated to the same linkage group for the purpose of examining potentially causal linked genes. These linkage groups are putative only as resolution for this test is limited by our population size. For a comparison with results for red rot, we performed a GWAS for TCH and CCS for all clones in the same population as screened and reported for red rot resistance.

Highly significant SNPs for red rot resistance were aligned to the sorghum genome in order to identify genes that were physically located next to or on top of these SNPs. Some details of the methods used are provided below.

### Identification of nearby genes

The genomic location of SNPs most significantly associated with red rot resistance, TCH and CCS was determined by searching the sugarcane and sorghum genomes on the CSIRO public genome browser (http://gbrowse-ext.bioinformatics.csiro.au /). Sorghum was used as a reference genome as it is the closest diploid relative to sugarcane with a high level of synteny between the two genomes, but is smaller with more detailed gene annotation (Garsmeur et al., 2018). Genes located at or within 5 kb of a significant SNP were investigated to determine their likely biological function. This was determined by observing their sequence homology to characterised genes, and their transcriptional regulation in sorghum using the Morokoshi sorghum transcriptome database (http://matsui-lab.riken.jp/morokoshi/Home.html).

### Genomic selection

Methods for genomic selection and prediction mostly followed those detailed by Deomano et al. (2020). For each trait, four Bayesian models and two Machine Learning methods were fitted to the data. The Bayesian models used were BayesA, BayesB, Bayesian Lasso (BL), and Genomic BLUP (GBLUP) (Crossa et al., 2010; Pérez and Campos, 2014). The Bayesian models differ on the assumed distribution of the clone effects. Three sets of explanatory variables per model were used, with these being pedigree (A), marker (M) and pedigree + marker (AM) information (Deomano et al., 2020). Pedigree data over 3 - 10 generations was retrieved from information on ancestors in a database owned by SRA. What was previously considered to be a semi-parametric method (Gianola et al., 2006), the Reproducing Kernel Hilbert Spaces (RKHS) is now also included in the Machine Learning (ML) group (Gonzalez-Recio et al., 2014). The A type of explanatory variable was used on RKHS. For both the M and AM type, the Reproducing Kernel Hilbert Spaces-Kernel Averaging (RKHS-KA) model was used (Gonzalez-Camacho et al., 2012). Random Forest (RF) (Breiman, 2001), one of the popular ML methods, which is a tree-based ensemble method for regression was fitted to the data using the A, M and AM explanatory variables. For all three sets of explanatory variables and 6 models, the full SNP data was fitted.

For the Bayesian models including RKHS the BGLR R-package (Pérez and Campos, 2014) was used with default values provided by the software. The number of MCMC iterations, burn in and thinning were 10K, 1K and 10, respectively. The ranger R-package was used for the RF model with ntree = 500, nodesize = 5 and other parameters set to default (Gonzalez-Camacho et al., 2018). Each model per type per trait was cross-validated on 50 replicates of a randomised 80 training:20 test dataset. The prediction accuracy of a model was calculated as the Pearson’s product-moment correlation coefficient between the observed trait and predicted trait for the test dataset. Accuracy was calculated for each replicate for each model per type per trait. Accuracies were then averaged across 50 replicates per model per type per trait.

The above methods were also applied for the M type models except with some markers with large effects identified from the GWAS analysis considered as fixed effects, and all other markers considered as random effects. Except for RFR, the second set of SNP data consisting of the remaining non-significant SNPs were fitted as random effects. For the RKHS model, only one kernel was used in the model.

## Results

### Analysis of variance and distribution of red rot resistance

**Figure 1:**
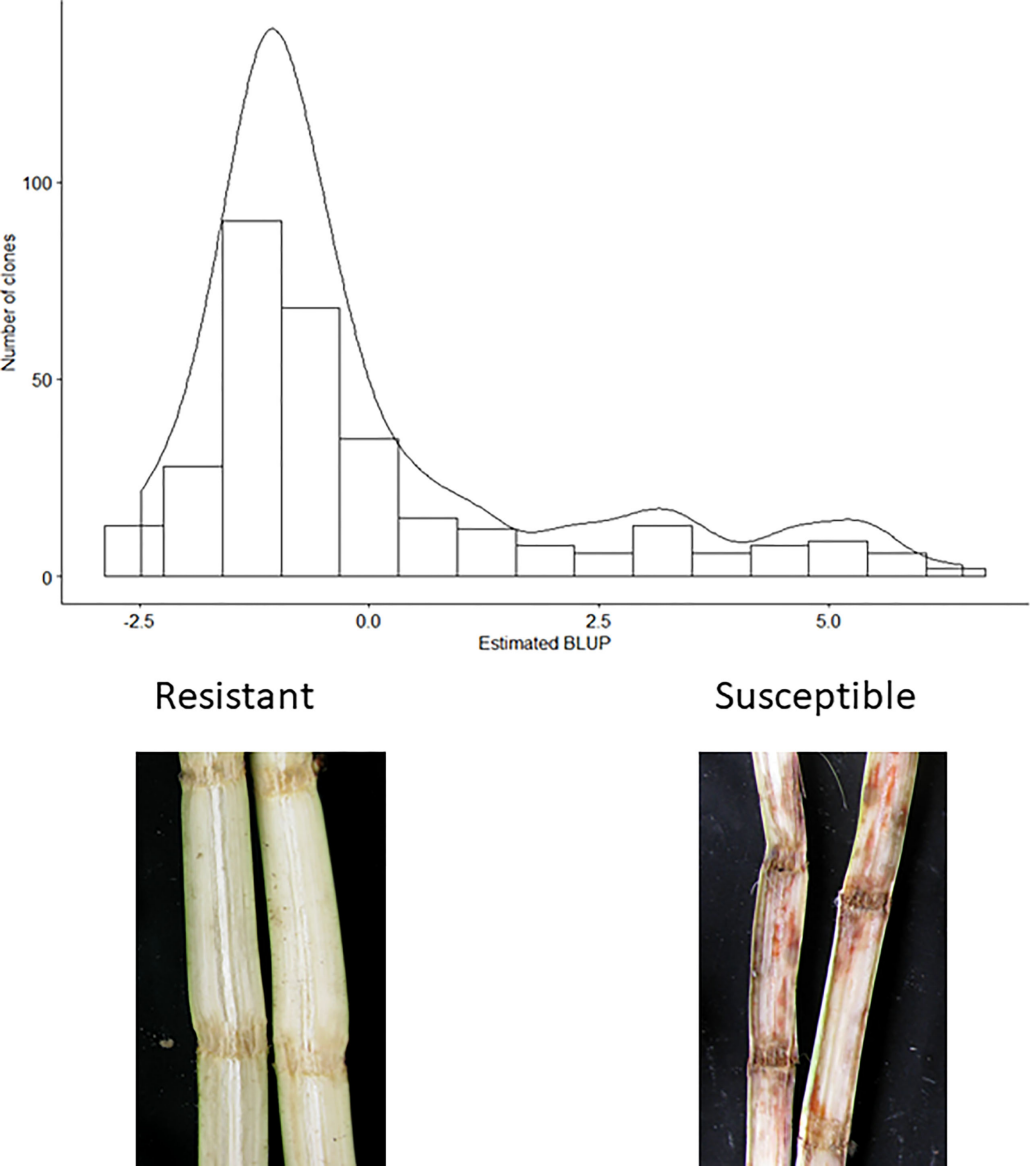
Distribution of estimated red rot BLUPs. Lowest BLUP values correspond to greater resistance levels. Photographs show typical symptoms observed in resistant and susceptible clones in the assay.

The broad-sense heritability for the scores for response to red rot was estimated to be 0.89, indicating nearly 90% of variation in measured phenotype was attributable to genetic effects (with the remainder due to experimental or environmental effects). The majority of clones appeared resistant or highly resistant to red rot in response to the screening method (**Figure 1**).

### Genome-wide association studies

**Figure 2:**
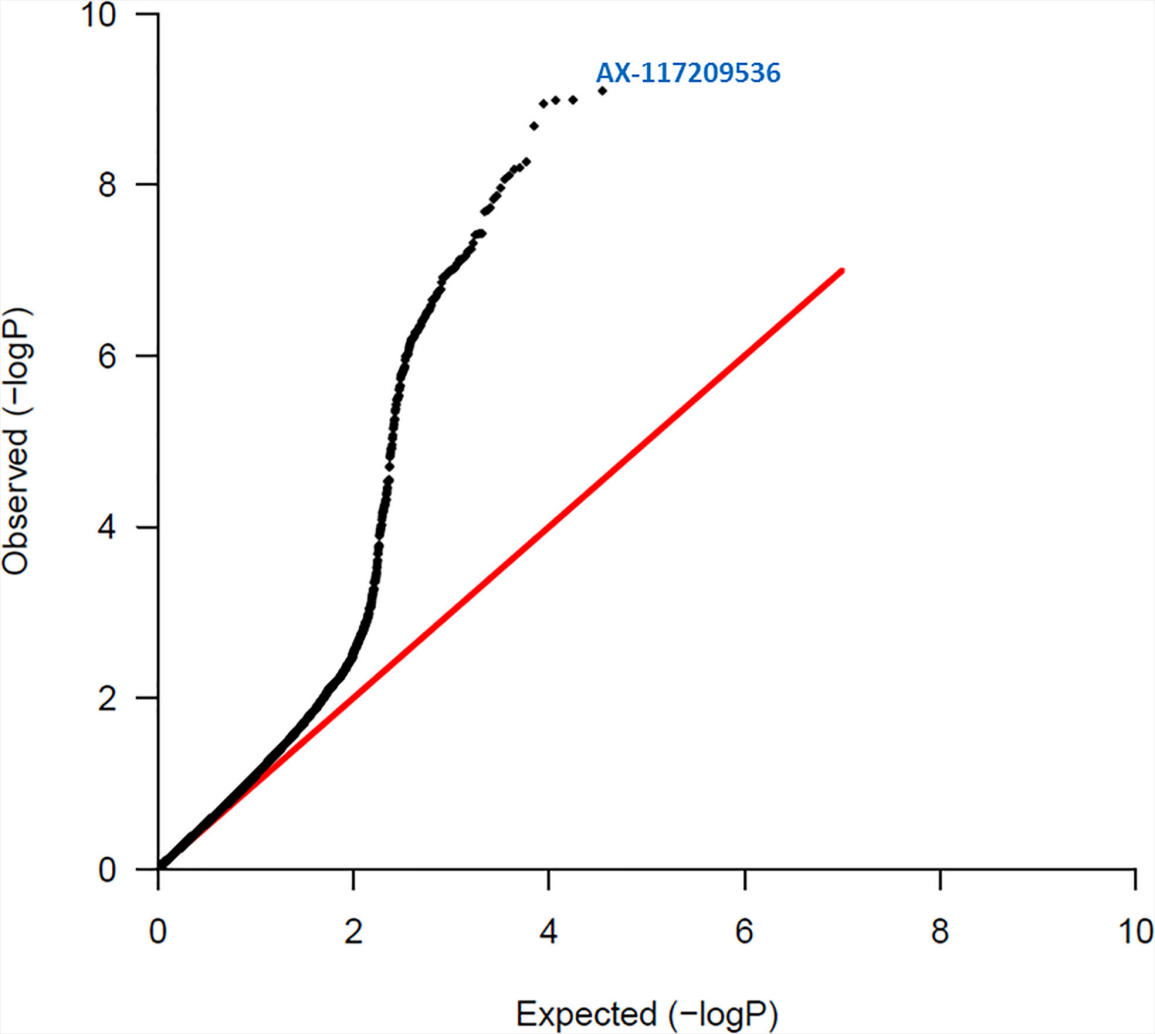
Quantile – Quantile plot of red rot SNPs. Expected normal distribution of p-values assuming no associations (null hypothesis) is given on the x axis, and the observed p-values on the y axis. The red line indicates the expected trend if observed p-values were normally distributed. The most significant SNP (AX-117209536) is indicated on the figure and was associated with red rot disease at p = 8.00 x 10^-10^.

A summary of the results of association tests between individual SNP markers and red rot resistance are given in **Table 1**. **Figure 2** shows a quantile-quantile plot of p-values for all SNPs in the red rot association mapping population. The observed p-values in our study deviated significantly from a normal distribution that would be expected by chance if there was no association with the trait. This is consistent with markers declared significant at the lowest P values having low false discovery rates (i.e. ratio of number of markers expected at the P value to be declared by random chance compared with number observed if the null hypothesis of no markers linked to the trait was valid, **Table 1**), meaning there is a high level of certainty that none of these markers are being declared as significant due to Type 1 statistical errors (random chance).

**Table 1:** GWAS results for all traits.

P value	By random chance	Red Rot resistance	Tonnes cane per hectare		Commercial cane sugar	
			P	1R	P	1R
**≤ 10^-2^**	352	714	907	1083	497	1037
**≤ 10^-3^**	35	243	184	232	63	134
**≤ 10^-4^**	4	184	42	47	3	23
**≤ 10^-5^**	0	141	10	4	0	0
**≤ 10^-6^**	0	99	0	0	0	0
**≤ 10^-7^**	0	35	0	0	0	0
**≤ 10^-8^**	0	10	0	0	0	0
**≤ 10^-9^**	0	1	0	0	0	0

The number of SNPs associated with Red Rot resistance, cane yield (TCH) and commercial cane sugar content (CCS) at different p values is shown, along with the number of SNPs expected to be associated with the trait by random chance (i.e. assuming the null hypothesis of no markers linked to red rot resistance). For TCH and CCS, the crop cycle in which the trait was measured is indicated where P, plant crop; 1R, first ratoon.

Thirty-five markers were found to be significantly associated with red rot at P ≤ 10^-7^, and this level of P corresponded to a near zero false discovery rate. These markers were all found to be in LD, with most in strong LD (r > 0.8), suggesting all were associated with a common QTL for resistance to red rot.

The number of markers associated with TCH and CCS at low P values (<10^-4^) was considerably less than for red rot, with no markers associated at P values less than 10^-6^. However, a higher number of markers than expected by random chance were associated with TCH and CCS in both the plant and first ratoon crops for p values between 0.001 and 0.01, with the ratoon crop having higher numbers. For TCH, a higher number of SNPs were also associated with the trait than expected by random chance for P values ≤ 0.0001 and ≤ 0.00001. This was also the case for CCS for P ≤ 0.0001 in the ratoon crop only. SNP associations for each of TCH and CCS traits were strongly correlated between plant and ratoon crop measurements, consistent with performance for each of these traits being correlated between crop cycles (data not shown). However, there was no correlation between SNP marker effects for TCH and CCS, indicating that these two traits appear governed by different genes.

### Location of significant SNPs on sorghum and sugarcane genomes, and nearby genes

**Figure 3:**
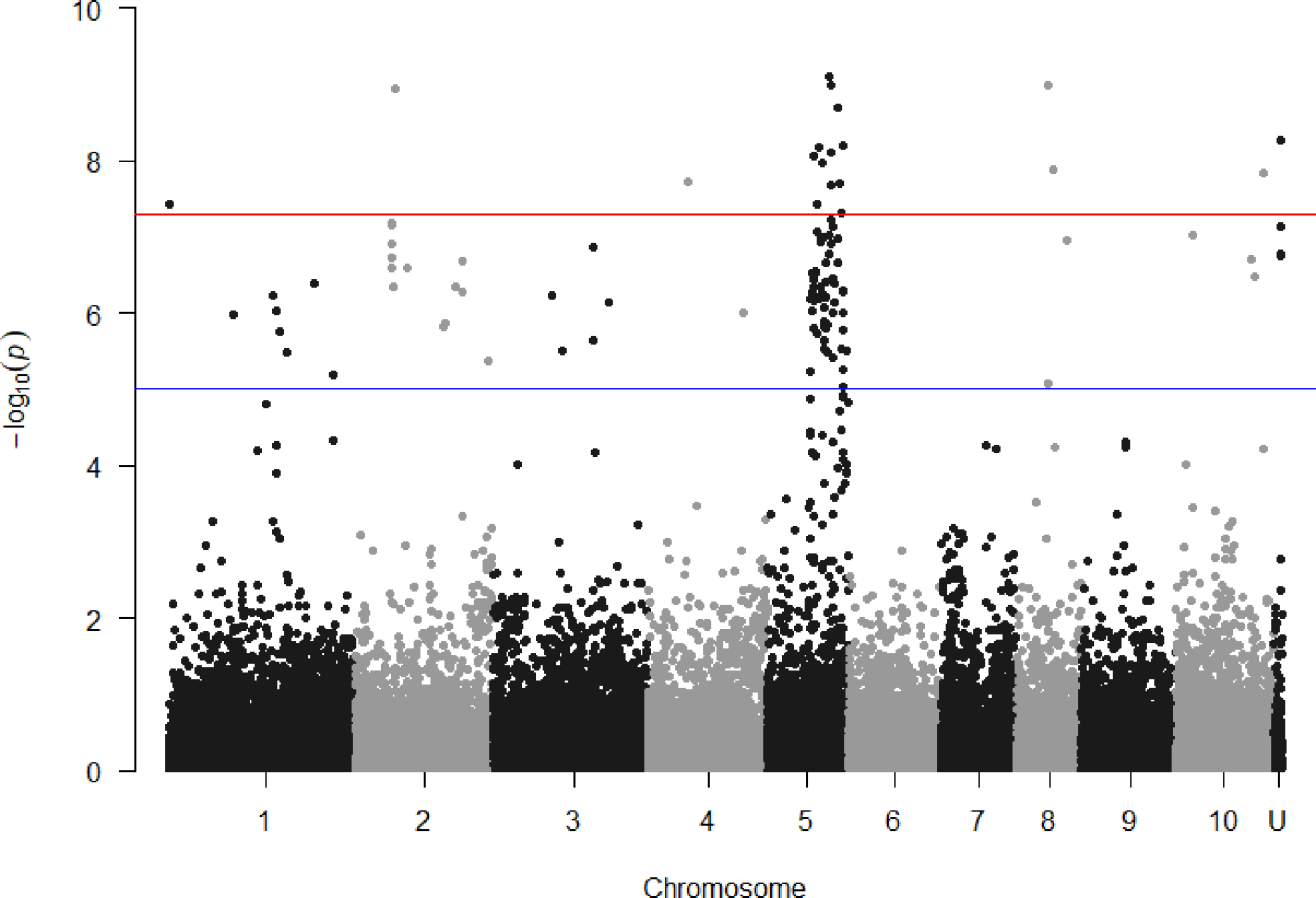
Manhattan plots showing P value of SNPs obtained from association analysis for red rot resistance score versus locational alignment to the sorghum genome (chromosomes numbered 1 to 10, U indicating no alignment found). For guidance, the red line indicates p = 5x10^-8^ and the blue line p = 1x10^-5^.

**Figure 4:**
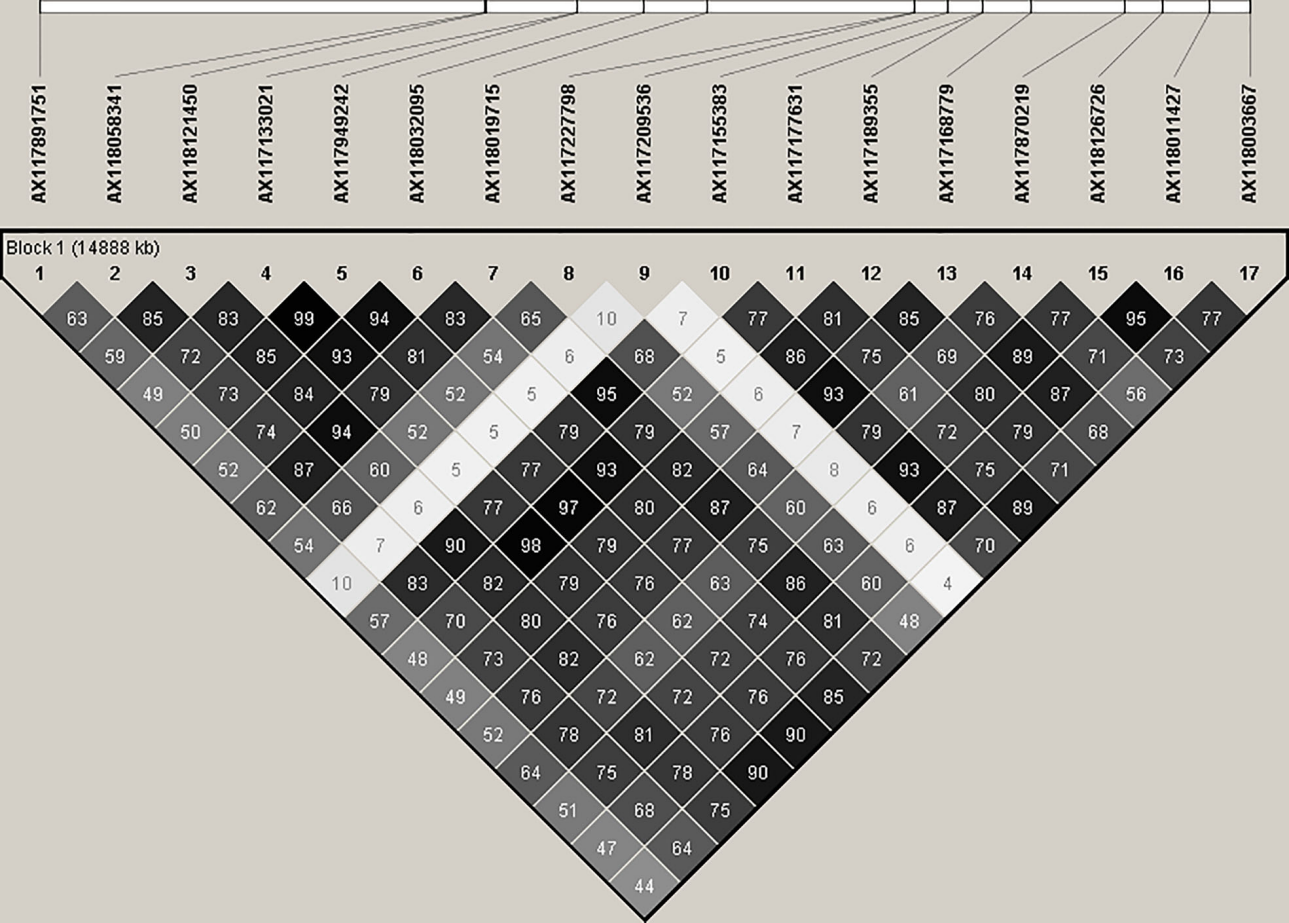
Linkage disequilibrium. r^2^ (expressed as %) observed between 17 significant SNP markers, and physical alignment to the sorghum genome. These SNPs are closely linked on the sorghum genome and are significantly (P< 10^-7^) associated with resistance to red rot.

SNPs associated with resistance to red rot at P< 10^-7^ were aligned with the sorghum genome, and those SNPs (29) with closely located genes (< 5kb from the SNP) were listed in **Table 2**. Many of the SNPs occurred within the coding sequence of genes, reflected in **Table 2** by the high proportion of genes located 0.0 kb from significant SNPs. Out of these 29 SNPs, most (21) were aligned to sorghum chromosome 5, and a smaller number were aligned to several other chromosomes (**Figure 3**). Of these, 17 SNPs corresponded to a 14.6 Mb section of sorghum chromosome 5 (**Figure 4**). All of these SNPs were in strong LD in the sugarcane population in this study apart from one, AX117209536, which was in weaker LD (although still statistically significant) with the others in this group (**Figure 4**). A weak LD in our breeding population among SNPs closely physically located on the genome could arise because of recombination in one or more key ancestors. In addition, sugarcane sorghum chromosomes 5, 6 and 7 are rearranged in some of the chromosomes inherited from *S. spontaneum* which could lead to incomplete synteny to sorghum in this region (Garsmeur et al., 2018).

**Table 2:** Genes co-located with red rot resistance associated SNPs.

SNP (AX#)	P value	Effect size	Nearby genes (Sobic.#)	kb from SNP	Gene family/function
117163812	3.73E-08	-0.8772	001G000400	0.0	Pleiotropic drug resistance protein.
117962959	1.13E-09	-0.9654	002G141200	0.0	DNA binding protein.
117995732	1.39E-07	0.8357	003G302400	0.0	Unknown protein.
117891751	8.65E-09	0.8897	005G126600	0.0	Auxin signalling F-box 2.
118058341	3.78E-08	0.8504	005G142900	0.0	Pentatricopeptide repeat (PPR) superfamily protein.
118121450	8.50E-08	0.8353	005G143300	0.0	Histone chaperone domain CHZ domain containing protein.
117949242	6.62E-09	-0.9233	005G149600	2.5	Cytochrome P450, family 76, subfamily C, polypeptide 2. Biotic stress inducible in Arabidopsis.
117315834	1.03E-07	-0.8287	005G153000	0.0	Agenet domain containing protein.
118032095	1.09E-08	-0.8979	005G154800	0.0	OsWAK receptor-like cytoplasmic kinase.
118019715	9.90E-08	0.8439	005G160600	0.5	OsFBO15 – F-box and other domain containing protein.
117191093	8.34E-08	-0.8368	005G162900	0.0	DNAJ heat shock N-terminal domain-containing protein.
117891837	1.01E-07	0.8397	005G163400	0.0	DNAJ heat shock N-terminal domain-containing protein. Biotic stress responsive in Arabidopsis.
117992398	1.67E-07	0.8141	005G176600	1.0	Eukaryotic aspartyl protease.
117209536	8.00E-10	0.9071	005G177800	0.0	Basic helix-loop-helix (bHLH) DNA-binding superfamily protein.
117272856	1.21E-07	0.8837	005G180900	1.0	DUF630/DUF632 domains containing protein, putative, expressed. bZIP transcription factor.
117155383	5.83E-08	0.8824	005G181000	0.0	Acyl-coenzyme A oxidase. Biotic stress responsive in Arabidopsis.
117133021	7.78E-09	-0.9088	005G182200	0.0	NB-ARC domain-containing disease resistance protein.
117177631	1.02E-09	-0.9732	005G183600	0.0	Mannose-binding lectin superfamily protein. Similar to Jasmonate-induced protein.
118058750	3.87E-08	-0.9076	005G186200	0.5	PATATIN-like protein 4.
117168779	7.38E-08	0.9070	005G189700	0.0	Expressed protein.
117870219	2.06E-09	0.9613	005G200300	1.0	Tyrosine aminotransferase.
118126726	1.99E-08	0.9474	005G204700	0.0	Pectin lyase fold/virulence factor domain containing protein.
118011427	4.81E-08	0.9321	005G209600	0.0	P-loop nucleoside triphosphate hydrolases superfamily protein with CH (Calponin Homology) domain.
117927589	1.03E-09	-0.9799	005G212800	2.0	Carboxyl-terminal peptidase, unknown function.
117301744	1.34E-08	0.8960	009G216700	0.0	WD domain, G-beta repeat domain containing protein.
117874758	9.39E-08	0.8523	010G054400	0.0	Leucine-rich repeat protein kinase family protein. Biotic stress responsive in Arabidopsis.
117154432	2.00E-07	0.8357	010G214500	0.0	Purple acid phosphatase.
117133579	5.39E-09	-0.9333	K004600	1.0	NADH:ubiquinone/plastoquinone oxidoreductase, chain 3 protein.
117879610	7.51E-08	-0.8435	K031500.1	0.0	DNA binding, ATP binding.

A distance from SNP of 0.0 kb indicates that the SNP occurs within the gene. Only highly significant SNPs (p ≤ 10^-6^) with closely co-located genes on the sorghum genome are listed in the table. Effect size indicates the difference between clones with the SNP and the clones that are homozygous and lack the SNP (in rating units).

### Genomic prediction

Accuracies of genomic prediction for red rot resistance, CCS and cane yield are presented in **Table 3**. The accuracies attained for red rot (up to 0.50) were greater than for the other traits. This is consistent with what may be expected considering that a higher number of markers were observed as associated with red rot at low p values than for the other traits.

**Table 3:** Genomic prediction accuracies per type, per model, per crop class and per trait.

Type	Model	Red Rot resistance		CCS		TCH	
		Random	Mixed	P	1R	P	1R
A	BayesA	0.25		0.20	0.19	0.11	0.16
A	BayesB	0.26		0.19	0.18	0.11	0.16
A	BL	0.25		0.18	0.18	0.11	0.16
A	GBLUP	0.24		0.22	0.22	0.11	0.16
A	RKHS	0.25		0.22	0.21	0.12	0.16
A	RFR	0.18		0.18	0.17	0.11	0.14
M	BayesA	0.40	0.15	0.27	0.27	0.20	0.22
M	BayesB	0.44	0.48	0.18	0.20	0.17	0.19
M	BL	0.41	0.50	0.28	0.28	0.20	0.22
M	GBLUP	0.41	0.50	0.28	0.28	0.20	0.22
M	RKHS	0.41	0.51	0.29	0.29	0.19	0.22
M	RFR	0.46	0.49	0.28	0.28	0.31	0.33
AM	BayesA	0.40		0.27	0.28	0.20	0.23
AM	BayesB	0.45		0.23	0.23	0.17	0.19
AM	BL	0.42		0.28	0.29	0.20	0.22
AM	GBLUP	0.40		0.28	0.28	0.18	0.22
AM	RKHS	0.40		0.28	0.28	0.17	0.21
AM	RFR	0.46		0.27	0.28	0.30	0.32

CCS, commercial cane sugar; TCH, tonnes cane per hectare; RR, red rot. The types of model refer to models (Bayes A, Bayes B, Bayesian Lasso, Ridge regression (GBLUP), Kernal Hilbert spaces (RKHS), Random Forest Regression (RFR), with pedigree data only (A), marker data only (M) and pedigree and marker data combined (AM). For Red Rot resistance, accuracies are given for models assuming random marker effects only (random) and models assuming a mixed model (Mixed), with 29 markers given in Table 3 designated as fixed effects and the remaining markers as random effects.

For red rot, the A type model (i.e. using just pedigree data without marker data) gave lower accuracy than the M and AM models (**Table 3**). This indicated the inclusion of DNA marker data in the model improved prediction of trait performance over and above that which can be attained through pedigree data alone.

In addition to “standard” genomic prediction models where markers are assumed as random effects, the 29 red rot SNPs listed in **Table 2** at P< 10^-7^ were added as fixed effects. Except for one case, the accuracies of prediction in most cases for the model with the fixed effects added were higher (0.48 to 0.51) than without (0.41-0.46). This result indicates adding large effect markers separately in the genomic prediction models can improve prediction capacity. However, there was a single exception to this result with the Bayes A model gave a very low accuracy when the fixed effects were added, and reasons for this were unclear.

### Effect of the QTL

**Figure 5:**
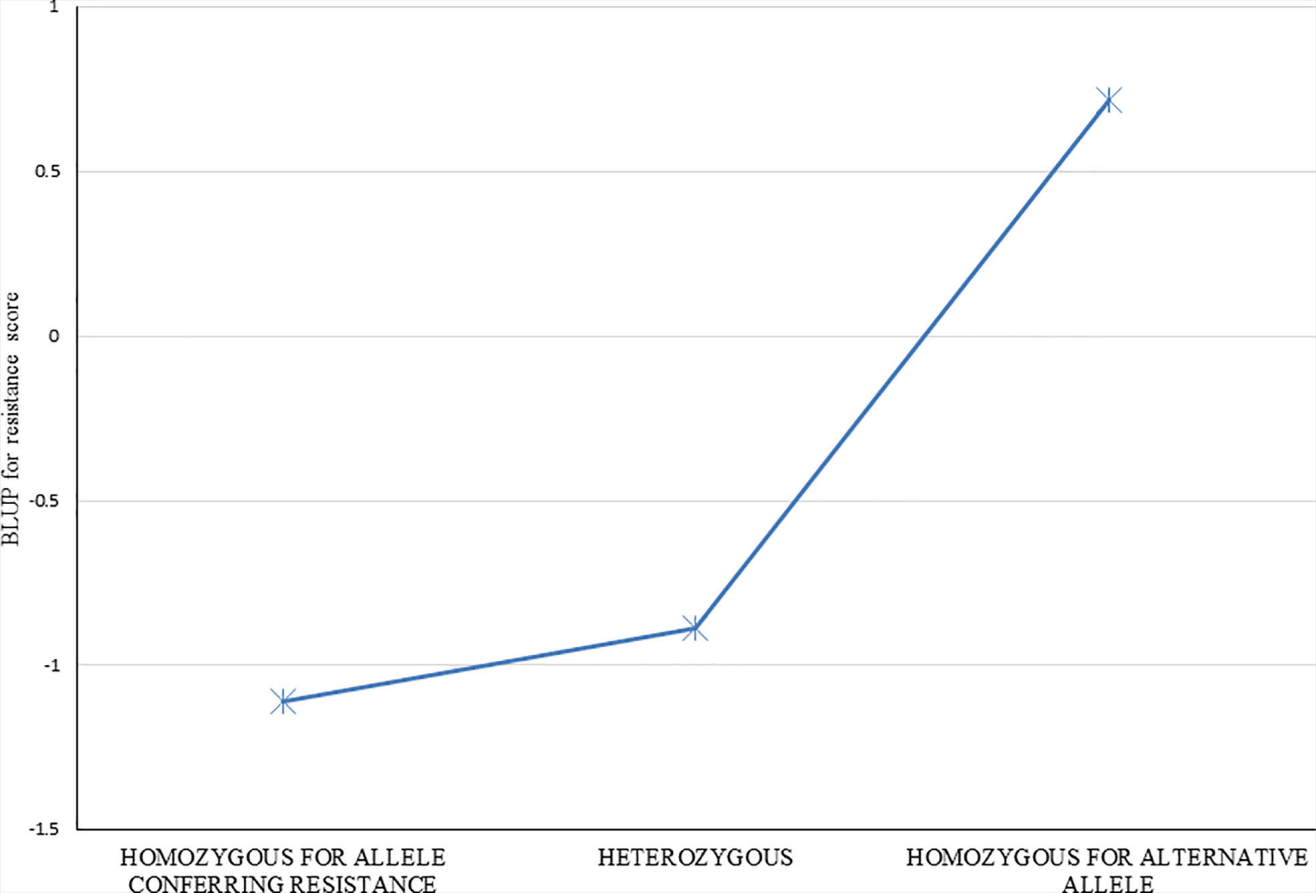
Resistance to red rot of groups of clones with the three different observed SNP marker genotypes (ie. Each of two homozygotes, and the heterozygote) for the markers found associated with red rot resistance at P<10^-7^. Average resistance score BLUPs for genotypes within each of the three groups were determined for each of the 35 markers individually, and the average of all 35 markers is shown.

Average resistance scores for clones homozygous for each allele, and heterozygous for the alleles were determined for each of 29 markers listed in **Table 2**. Effects for each of the individual 29 markers were very similar (data not shown), as expected given the strong LD (correlation) among this group of markers, and thus similar to the overall average of all markers (**Figure 5**). Clones with the resistance allele were more resistant by about 1.5 resistance rating units than clones without the resistance allele. A strong dominance effect of the resistance allele is also apparent, with the average resistance of the heterozygous clones being similar to that for clones homozygous for the allele conferring resistance (**Figure 5**). This result suggests that the QTL (or multiple closely linked QTL) linked with this group of SNP markers has both an additive and dominance variation component.

**Figure 6:**
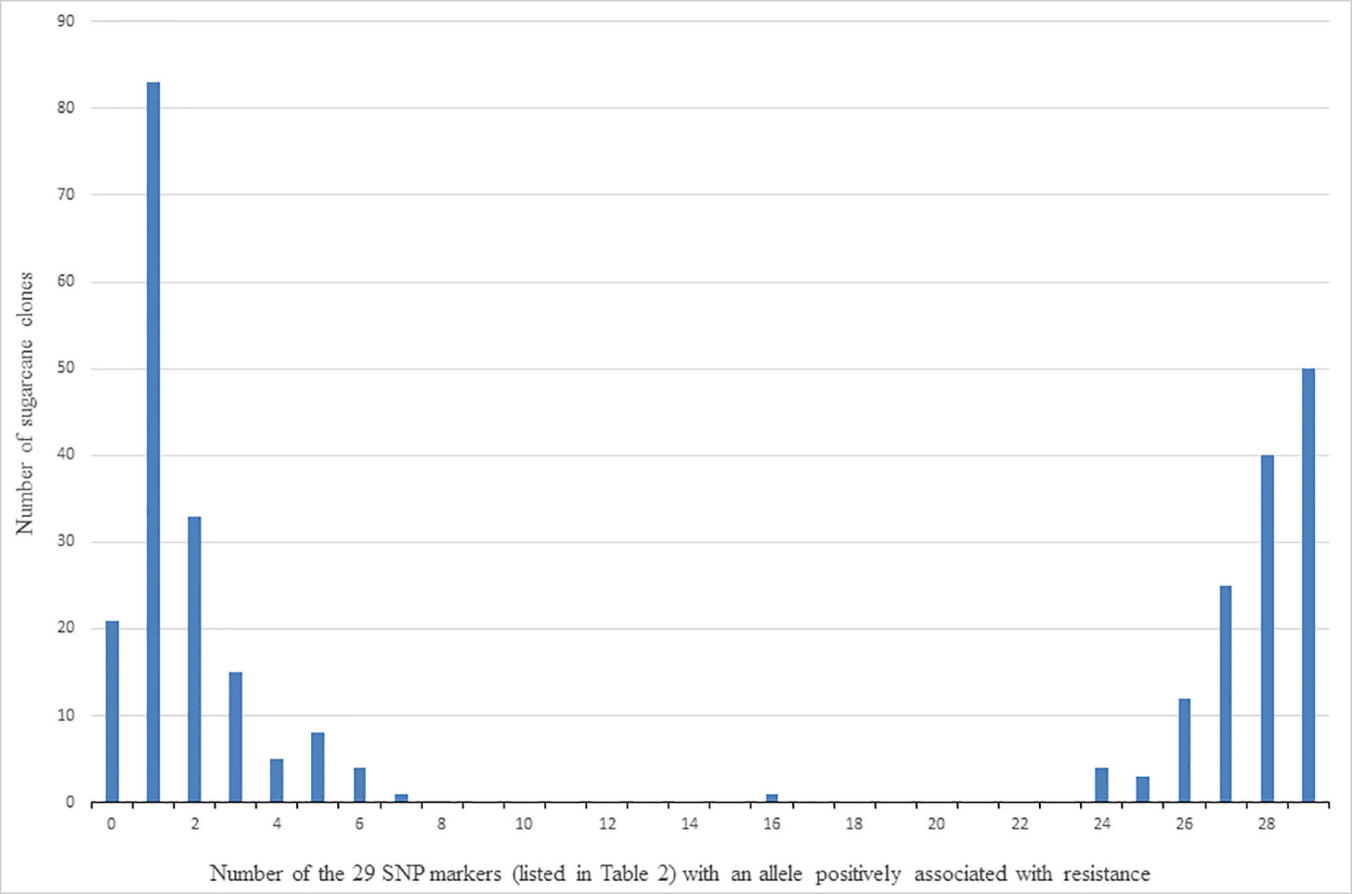
Number of clones (out of 305) versus number of the 29 SNP markers listed in **Table 2** that detected the allele (for each marker) associated with resistance to red rot.

Clones were classed into those putatively with and without the QTL (or multiple QTLs) characterised by the presence of alleles of the 29 linked SNP markers in **Table 2**, as follows. The allelic composition of each clone was firstly determined in terms of the number of the 29 SNP markers for which it had at least one copy of the allele found positively associated with resistance to red rot. The number of clones with different numbers (ranging from zero to 29) of SNP markers with at least one copy of the positively associated allele is shown in **Figure 6**. For example, this shows there are 82 clones for which only one of the 29 SNP markers presented with the allele positively associated with red rot resistance. Because of the high linkage disequilibrium among this set of markers, the clones are distributed as two contrasting and distinct groups. This consisted of one group of 170 clones (on the left side of **Figure 6**) having most of the 29 SNP markers not presenting with the allele associated with resistance (i.e. most markers SNP presenting as homozygous for the alternative alleles to the alleles associated with resistance), and another group (on the right side of **Figure 6**) having most of the SNP markers presenting with the allele positively associated with resistance (either as being heterozygous for the two alternative SNP alleles or homozygous for the SNP allele positively associated with resistance). Based on these results, the clones on the right-hand side of **Figure 6** (with most of the 29 SNP markers) presenting with at least one copy of the resistance allele) were arbitrarily classed for the purpose of further investigation as having the QTL associated with red rot resistance, while those on the left-hand side were considered to not have the QTL.

As shown in **Figure 5**, clones were arbitrarily classed into three resistance groups, as (i) susceptible, (ii) intermediate, and (iii) resistant, based on having a BLUP for resistance score of >2, 2 to 0, and<0, respectively. These classifications were cross-tabulated with presence or absence of the putative QTLs for resistance as defined above (**Table 4**). Of the one hundred and thirty-five clones with the resistance QTLs, 126 were resistant to red rot, and only two were classed as susceptible (**Table 4**). By contrast, there were 51 clones that were classed as susceptible, 49 of these did not have the resistance QTLs (**Table 4**). However, there were also 91 clones that were classed as being resistant but without the QTLs, which indicates that some other genetic factors could also contribute to resistance. In summary, these results are consistent with the resistance QTL (linked to the cluster of 21 (16 high LD) markers in **Table 2** having a penetrative, dominant effect on resistance, but with some other genetic factors present on other chromosomes also independently imparting resistance.

**Table 4:** Numbers of clones at different levels of resistance and whether a QTL for resistance to red rot was putatively present.

Resistance level	Resistance QTL present?		Grand Total
	No	Yes	
Susceptible	49	2	51
Intermediate	30	7	37
Resistant	91	126	217
Grand Total	170	135	305

## Discussion

The results indicate that a major QTL affects resistance to red rot within germplasm generated in the Australian sugarcane breeding programs. The presence of this effect, indicated by a cluster of closely linked SNP markers, appears to provide a high chance of resistance, with only a very small proportion (2% or less) of clones with resistance alleles linked to this QTL showing susceptibility. However, the presence of resistance in over 50% of clones without this QTL also indicates that other genetic factors also contributed to red rot resistance in the germplasm studied, reducing somewhat the overall difference in resistance levels of clones with the QTL versus those without, across the whole population.

The set of clones sampled for this study represented those routinely generated in the commercial sugarcane breeding program in Australia. The observation that less than 20% of the sampled clones were susceptible to red rot, with most clones exhibiting resistance, is consistent with the situation seen for many years in the Australian sugarcane breeding program and industry, where susceptibility to red rot is not generally an important problem. However, this situation is clearly different to other countries, such as southern Asian countries where cultivar susceptibility to red rot is a critical problem, and it is of interest to understand possible reasons for the difference. It is at present unclear if the potentially important QTL conferring resistance to red rot in the germplasm, or other genetic effects, contributes to the high proportion of resistant clones relative to the situation in other countries, or if pathogen variation is the major cause of differences. It is of interest to understand if the major QTL in the Australian germplasm exists in sugarcane cultivars and breeding programs in countries such as India. At this stage, the value of this QTL in other countries is unknown, and its effectiveness (or vulnerability) against different evolved strains of red rot in other countries should be investigated. The use of common SNP markers available for easily comparing germplasm in different programs will facilitate this investigation in the future. For the Australian sugarcane breeding program, based on the results in this study, it would be possible to screen for several of the SNP markers (listed in **Table 2**) linked to the major resistance QTL to ensure elimination of red rot susceptible material from any selection populations. In addition, selected SNPs for red rot resistance could possibly be usefully included in a targeted, low-cost marker platform used to screen sugarcane clones for multiple disease traits.

The pattern of results shown in **Table 4** suggests some degree of non-additive genetic effects may arise, which could limit prediction of resistance using models based on only additive genetic effects, including the standard GWAS methods and most of the well-established genomic prediction models used in this study. In this situation, models based on decision trees may be more effective in predicting resistance. For example, a decision tree that is based on an initial branch that predicts a clone is relatively resistant if it had a set of alleles associated with resistance for the majority of the SNP loci indicated in **Table 2**, may be appropriate. This may be one reason why the random forest method of genomic prediction produced slightly better accuracy levels than the other methods (where the QTL effect was not included as a separate fixed effect) (**Table 3**).

A cluster of SNPs strongly associated with resistance to red rot were located within a 14.6 Mb region of sorghum chromosome 5, suggesting the identification of a novel QTL. There is an indication from the SNP effect that minor QTL are also present on alternative copies of this chromosome in the sugarcane genome. This QTL region has not to our knowledge been previously reported. Singh et al. (2016) used association mapping to identify putative red rot responsive sugarcane QTLs homologous to regions of chromosome 2 and 7 in sorghum. These effects appeared smaller than those identified in our study, although resolution of these was also limited by small population size. Sathyabhama et al. (2015) identified differentially expressed EST clusters in red rot resistant sugarcane variety Co 93009 with sequences that were homologous to regions of sorghum chromosomes 1, 3, 4, 7, 8, and 9.

Many of the genes co-located with SNPs linked to the proposed QTL in this study (and listed in **Table 2**) encode proteins involved in plant response to pathogens. These include jasmonate induced proteins, pleiotropic drug resistance proteins (a general defence protein) (Sasabe et al., 2002), pectin lyase fold/virulence factor domain containing proteins, DUF630/DUF632 domains containing bZIP transcription factors (involved in pathogen response) (Alves et al., 2013), auxin signalling F-box 2 proteins (Navarro et al., 2006), and NB-ARC domain-containing disease resistance proteins. We have corroborated the expression of some co-located genes at the transcript and protein level. A significant SNP (AX 117962959) which was found on a DNA binding protein gene, and its peptide was present in the proteome developed during *C. falcatum* sugarcane interaction. An RNA binding/nucleic acid binding/zinc ion binding protein was associated with red rot resistance and found in this proteome (Kumar et al., 2020). Another significant SNP (AX 117891751) was located in Auxin signalling F-box 2 gene. A previous study showed differential expression of a F-box domain containing protein in a subtractive library (Sathyabhama et al., 2015) and in the proteome (Kumar et al., 2020). Prathima et al. (2013) also found its expression through differential display (DD-RT-PCR) in a resistant variety after pathogen inoculation. Specific expression of a set of chitinases was demonstrated in a resistant variety as well as in a susceptible variety tolerating red rot development due to plant growth-promoting rhizobacteria (PGPR) mediated induced systemic resistance (Viswanathan et al., 2003; Viswanathan et al., 2009). Subsequently, Rahul et al. (2016) demonstrated that sugarcane chitinase genes were upregulated in sugarcane cells when they are challenged with *C. falcatum* elicitors, and some success has been achieved in supressing red rot symptoms in sugarcane by transgenic overexpression of chitinases (Tariq et al., 2018) or by application of biocontrol agents that produce chitinases (Joshi et al., 2019). Further investigation of this region of the sugarcane genome may provide greater insights into the mechanisms underlying genetic control of red rot resistance in sugarcane.

Significant SNPs were identified in a cytochrome P450 gene which showed up regulation in a resistant variety (Prathima et al., 2013). The SNPs (AX118019715, AX117191093, AX117891837) are located at genes which showed specific expression in a resistant variety (Co 93009) after pathogen inoculation in proteomic studies (Kumar et al., 2020). The SNP (AX117133021) was co-located on a disease resistance gene and specific expression of disease resistant protein RPM1 and RPS5 was established in a resistant sugarcane variety Co 93009 (Viswanathan et al., 2016). For SNP (AX117874758), increased expression of its co-located gene has been found in a resistant variety (Sathyabhama et al., 2015). Using differential display (DD)-RT-PCR), LRR family protein expression was found to increase in resistant variety (Co 93009) after pathogen inoculation and in sugarcane suspension cultures treated with *C. falcatum* elicitors (Prathima et al., 2013; Rahul et al., 2016). The SNP co-located to the gene NADH ubiquinone oxidoreductase (SNP ID AX117133579) was also identified in a resistant variety in a gene expression study (Rahul et al., 2016).

The 14.6 Mb region homologous to the sorghum genome in **Figure 3** also contains genes which have orthologues reportedly transcriptionally regulated in response to stress when tested in the model plant *Arabidopsis thaliana*. This includes a number of genes associated with fungal pathogen defence including a cluster of chitinase A genes (Sobic.005G177100, Sobic.005G177400, Sobic.005G177500, Sobic.005G177600) whose orthologues are transcriptionally activated in response to abiotic stress in Arabidopsis (Berardini et al., 2015). Interestingly, the single most significant SNP in our study (AX117209536, p = 8.00E-10) was also the closest SNP to this cluster, located just 13 kb away.

At this stage the ancestral origin of the major QTL identified in this study is not known. Markers with LD ≥0.8 are considered to be in strong LD and **Figure 4** shows clusters of markers in strong LD which probably correspond to ancestral haplotypes segregating as large blocks in the population of sugarcane cultivars screened. This cluster appears to correspond to a homolog of sorghum chromosome 5. It is known from other mapping studies and cytogenetic analysis there are from 10-12 homologous copies of every chromosome in sugarcane (Aitken et al., 2005; Piperidis and D’Hont, 2020). Wei et al. (2007) detected LD in a collection of sugarcane cultivars and identified haplotype blocks that contained from 2 to 10 markers although high significance levels were needed to reduce spurious associations. They identified significant LD between markers up to 40 cM apart but the majority of LD occurred between 0 and 30 cM. In this study, when four of the markers associated with red rot were also mapped to a single linkage group aligned to sorghum chromosome 5 of a genetic map of variety Q208, they covered 33.7 cM (K Aitken pers comm.). This is consistent with the LD previously identified in sugarcane and its breeding history which has a strong foundation bottleneck (Wei et al., 2007).

Results for cane yield and CCS on the same materials in this study provided an interesting benchmark for the results for red rot resistance. The number of markers observed for association with red rot resistance was much higher, and P values lower, than for cane yield and CCS, consistent with larger additive marker effects for red rot resistance. The genomic prediction accuracies for TCH and CCS in the population reported here were low (< 0.25) because of the relatively small number of clones observed (307). These accuracies are lower than those found with a larger number of clones (about 2500) measured in prior work (with accuracies of >0.35) (Deomano et al., 2020). However, a significantly higher number of markers were observed as being significantly associated with both cane yield and CCS than expected by random chance, indicating that markers are explaining a proportion of variation observed and indicative of the value of the extra data collected. It is likely that greater accuracy and resolution of smaller marker effects linked to red rot resistance could be attained with a larger population than the 305 clones used in the current study.

## Data availability statement

The data presented in the study are deposited in the GitHub repository, accession number https://github.com/SugarAus/data.git.

## Author contributions

AO’C, JD, PL, ED, XW, PJ, and KA performed the experiments and/or analysed data. PJ, PL, and RM conceived and designed the project. HG, KM, RV, and BR contributed to methodology development and related technical expertise. All authors contributed to the article and approved the submitted version.

## Funding

This project was supported by the Australian Government under the Australia-India Strategic Research Fund grant number 48454, and by Sugar Research Australia, Brisbane and the ICAR Sugarcane Breeding Institute, Coimbatore, India.

## Conflict of interest

Authors AO'C, JD, ED, XW and PL were employed by the company Sugar Research Australia Limited.

The remaining authors declare that the research was conducted in the absence of any commercial or financial relationships that could be construed as a potential conflict of interest.

## Publisher’s note

All claims expressed in this article are solely those of the authors and do not necessarily represent those of their affiliated organizations, or those of the publisher, the editors and the reviewers. Any product that may be evaluated in this article, or claim that may be made by its manufacturer, is not guaranteed or endorsed by the publisher.
