# Supplementary material for: Combining genomic selection with genome-wide association analysis identified a large-effect QTL and improved selection for red rot resistance in sugarcane
Source: Front Plant Sci. 2022 Oct 31;13:1021182. doi: 10.3389/fpls.2022.1021182 (PMC9660812; doi:10.3389/fpls.2022.1021182)
Supplement: Supplementary file 1 [file DataSheet_1.docx]

**Supplementary tables**

**Table S1: Genes co-located with SNPs significantly associated with TCH (plant crop**). A distance of 0.0 kb indicates that the SNP occurs within the gene sequence. Coeff indicates the effect size in Tonnes cane per hectare associated with the SNP. Only highly significant SNPs with closely co-located genes are listed in the table.

| **SNP  (* common in P and 1R)** | **P value** | **Effect size** | **Sorghum orthologue** | **Distance from SNP** | **Gene name/product** |
| --- | --- | --- | --- | --- | --- |
| AX117155904* | 1.83E-05 | -4.5757 | Sobic.001G234200 | 0.0 kb | Actin-related protein 7. |
| AX117306642* | 4.69E-06 | 5.1850 | Sobic.001G367500 | 0.0 kb | Arginyl-tRNA synthetase. |
| AX117919435 | 7.05E-05 | -3.6361 | Sobic.001G377300 | 0.0 kb | Phylloplanin. |
| AX118026274 | 3.09E-05 | -3.8601 | Sobic.001G377400 | 0.0 kb | *Zea mays* clone 483426 hypothetical protein. |
| AX117217989 | 5.70E-05 | -3.7896 | Sobic.001G379500 | 0.0 kb | WEB family protein. |
| AX117312817 | 1.14E-05 | 4.7379 | Sobic.001G465700 | 0.0 kb | Dethiobiotin synthetase. |
| AX118039275* | 2.80E-05 | 4.2015 | Sobic.001G473200 | 0.0 kb | Non-specific lipid-transfer protein-like. |
|  |  |  | Sobic.001G473300 | 0.0 kb | Protein pyrophosphatase mRNA. |
| AX117264019 | 1.48E-05 | 4.8265 | Sobic.002G017300 | 0.0 kb | Uncharacterised protein. |
| AX117304238 | 1.30E-05 | 4.7540 | Sobic.002G017300 | 0.0 kb | Uncharacterised protein. |
| AX117271031* | 2.87E-06 | 4.8563 | Sobic.002G271200 | 0.0 kb | UDP-glucose 4-epimerase 3. |
| AX117148131 | 2.96E-05 | -5.2194 | Sobic.003G135100 | 0.0 kb | Mediator of RNA polymerase II transcription subunit 26b. |
| AX117213520* | 7.26E-05 | 3.8938 | Sobic.004G085900 | 0.0 kb | Disease resistance protein (CC-NBS-LRR class) family. |
| AX117953446 | 9.35E-05 | -5.5979 | Sobic.004G119100 | 0.3 kb | Optic atrophy 3 (OPA3) like protein. |
| AX117310607 | 5.97E-05 | 4.1603 | Sobic.006G020100 | 0.0 kb | Uncharacterised protein. |
| AX117197744 | 1.57E-05 | 4.3294 | Sobic.006G035500 | 0.1 kb | Putative oxidoreductase. |
| AX117151814* | 4.69E-06 | -5.7233 | Sobic.008G113600 | 0.0 kb | Glutathione synthetase 2. |
| AX117256983 | 4.73E-05 | 4.2360 | Sobic.008G114300 | 0.0 kb | Acetyl-CoA carboxylase 1. |
| AX117312296 | 5.46E-05 | 3.9769 | Sobic.008G115000 | 0.0 kb | VIN3-like protein 1. |
| AX117172740* | 1.42E-06 | 4.9576 | Sobic.008G119800 | 0.0 kb | Putative disease resistance protein RGA1. |
| AX117986437* | 4.87E-05 | 4.4290 | Sobic.008G120200 | 0.0 kb | Uncharacterised protein. |
| AX117905240* | 1.83E-05 | -5.2407 | Sobic.008G133500 | 0.0 kb | Diaminopimelate epimerase. |
| AX118191010* | 1.02E-05 | -5.4053 | Sobic.008G134800 | 0.0 kb | Inactive shikimate kinase like 1. |
| AX117236342* | 2.10E-05 | 4.5593 | Sobic.008G136700 | 0.1 kb | Uncharacterised protein. |
| AX117960272 | 5.68E-05 | -4.7105 | Sobic.008G140700 | 0.0 kb | Polyadenylate-binding protein 7. |
| AX117164335 | 8.79E-05 | 4.0879 | Sobic.008G143700 | 6.0 kb | Metal transporter Nramp6. |
| AX118057772* | 2.43E-06 | -5.8067 | Sobic.008G152800 | 0.0 kb | Calcineurin B-like protein 2 . |
| AX117911855 | 2.55E-05 | 3.9746 | Sobic.008G152800 | 0.0 kb | Calcineurin B-like protein 2. |
|  |  |  | Sobic.008G152900 | 5.0 kb | TRAF-like superfamily protein. |
| AX117206568* | 2.05E-05 | -5.3165 | Sobic.008G156300 | 0.0 kb | Pyridoxal kinase (SOS4 gene). |
| AX117231750* | 1.87E-06 | 4.5231 | Sobic.008G159700 | 0.0 kb | ROOT HAIR DEFECTIVE 3 homolog 1. |
| AX117153102 | 9.19E-05 | 4.0890 | Sobic.008G164700 | 0.0 kb | RRNAD1 protein. |
| AX117202922* | 6.08E-06 | -4.7888 | Sobic.008G169400 | 0.0 kb | Auxin response factor 25. |
| AX117881055* | 2.55E-05 | 4.3101 | Sobic.008G170000 | 0.0 kb | Mediator of RNA polymerase II transcription subunit 19a-like. |
| AX117251632 | 5.32E-05 | -4.3034 | Sobic.008G170600 | 0.0 kb | Pentatricopeptide repeat-containing protein. |
| AX117236220* | 5.57E-06 | -5.4816 | Sobic.008G175000 | 0.0 kb | Putative receptor kinase. |
| AX118016411 | 9.54E-06 | -5.9903 | Sobic.008G175000 | 0.0 kb | LRR receptor-like serine/threonine-protein kinase. |
| AX117242235 | 4.09E-06 | 4.7638 | Sobic.008G190100 | 0.0 kb | Acetyl-CoA acetyltransferase. |

**Table S2: Genes co-located with SNPs significantly associated with TCH (ratoon crop).** A distance of 0.0 kb indicates that the SNP occurs within the gene sequence. Only highly significant SNPs with closely co-located genes are listed in the table.

| **SNP**  **(* common in P and 1R)** | **P value** | **Sorghum orthologue** | **Distance from SNP** | **Gene name/product** |
| --- | --- | --- | --- | --- |
| AX117975219 | 7.03E-05 | Sobic.001G090800 | 0.0 kb | PHD finger family protein. Acyl-CoA N-acyltransferase with RING/FYVE/PHD-type zinc finger. |
| AX117208583 | 1.16E-05 | Sobic.001G096300 | 0.0 kb | Deoxyhypusine synthase. Drought responsive. |
| AX117280932 | 2.90E-06 | Sobic.001G104600 | 0.0 kb | Prostamide/prostaglandin F synthase.Thioredoxin superfamily. |
| AX117889972 | 9.48E-05 | Sobic.001G107300 | 0.0 kb | WD repeat-containing protein 5.Transducin/WD40 repeat-like superfamily protein. |
| AX117909950 | 4.68E-05 | Sobic.001G134300 | 0.0 kb | Uncharacterized. Tetratricopeptide repeat (TPR)-like superfamily protein. |
| AX118039690 | 5.17E-05 | Sobic.001G143600 | 0.0 kb | Sec7 family. Pattern formation protein EMB30. GDP/GTP exchange factor. |
| AX117287957 | 4.42E-05 | Sobic.001G144600 | 0.0 kb | Parafibromin. |
| AX117923778 | 5.19E-05 | Sobic.001G146800 | 0.0 kb | Uncharacterized (protein coding). |
| AX118017678 | 1.04E-05 | Sobic.001G149700 | 0.0 kb | Membrin-11. Vesicle transport v-SNARE protein. |
| AX118043623 | 1.13E-05 |  |  |  |
| AX117155904* | 1.25E-06 | Sobic.001G234200 | 0.0 kb | Actin-related protein 7. |
| AX117207819 | 3.58E-05 | Sobic.001G345100 | 0.0 kb | 1-phosphatidylinositol-4-phosphate 5-kinase/ zinc ion binding protein. |
| AX117306642* | 9.74E-06 | Sobic.001G367500 | 0.0 kb | Arginyl-tRNA synthetase. |
| AX118039275* | 6.94E-05 | Sobic.001G473200 | 0.0 kb | Bifunctional inhibitor/lipid-transfer protein/seed storage 2S albumin superfamily protein. |
|  |  | Sobic.001G473300 | 0.0 kb | Stage II sporulation protein. Protein phosphatase 2C family protein. |
| AX117271031* | 8.74E-05 | Sobic.002G271200 | 0.0 kb | UDP-glucose 4-epimerase. UDP-D-glucose/UDP-D-galactose 4-epimerase 1. |
| AX117246482 | 6.29E-05 | Sobic.003G036800 | 0.0 kb | WD domain, G-beta repeat domain containing protein. Transducin/WD40 repeat-like superfamily protein. |
| AX117880293 | 5.60E-05 | Sobic.004G081800 | 0.0 kb | Ubiquitin family domain containing protein. |
|  |  | Sobic.004G081900 | 1.0 kb | Subtilisin-like serine endopeptidase family protein. |
| AX117213520* | 7.46E-05 | Sobic.004G085900 | 0.0 kb | NB-ARC domain containing protein. |
| AX117873485 | 8.49E-05 | Sobic.004G102000 | 0.0 kb | Unknown function. |
| AX117178156 | 3.11E-05 | Sobic.004G221000 | 0.0 kb | Cyclic nucleotide-gated ion channel protein. |
| AX118118125 | 5.79E-05 | Sobic.005G191900 | 0.0 kb | Tonoplast monosaccharide transporter 2 family protein. |
| AX117201429 | 1.77E-05 | Sobic.008G089500 | 0.0 kb | Quinolinate synthase. |
| AX117151814* | 1.40E-05 | Sobic.008G113600 | 0.0 kb | Glutathione synthetase 2. |
| AX117172740* | 6.01E-06 | Sobic.008G119800 | 0.0 kb | NB-ARC domain containing disease resistance protein. |
| AX117986437* | 5.73E-05 | Sobic.008G120200 | 0.0 kb | Uncharacterized protein. |
| AX117905240* | 1.71E-05 | Sobic.008G133500 | 0.0 kb | Diaminopimelate epimerase family protein. |
| AX118191010* | 1.48E-05 | Sobic.008G134800 | 0.0 kb | Shikimate kinase family. |
| AX117236342* | 1.43E-05 | Sobic.008G136700 | 0.1 kb | Uncharacterized protein. |
| AX118057772* | 1.48E-05 | Sobic.008G152800 | 0.0 kb | CIPK family protein. |
|  | 1.48E-05 | Sobic.008G152900 | 5.0 kb | TRAF-like superfamily protein. |
| AX117206568* | 9.44E-05 | Sobic.008G156300 | 0.0 kb | Pyridoxal kinase (SOS4 gene) kinase. FkB-like carbohydrate kinase family protein. |
| AX117231750* | 1.18E-05 | Sobic.008G159700 | 0.0 kb | SEY1, putative, expressed. Root hair defective 3 GTP-binding protein (RHD3). |
| AX117202922* | 2.38E-05 | Sobic.008G169400 | 0.0 kb | ARF gene family. Auxin response factor. |
| AX117881055* | 3.50E-05 | Sobic.008G170000 | 0.0 kb | Mediator of RNA polymerase II transcription subunit 19a-like. Nucleolar protein NOP5. |
| AX117236220* | 3.44E-05 | Sobic.008G175000 | 0.0 kb | Receptor kinase. Leucine-rich repeat (LRR) protein kinase. |
| AX117216610 | 3.78E-05 | Sobic.009G024600 | 1.3 kb | Ethylene-responsive transcription factor RAP2-7. AP2 domain containing protein. |
| AX117148375 | 6.64E-05 | Sobic.009G119000 | 0.0 kb | Tetratricopeptide repeat containing protein. Heat shock protein DNAJ. |
| AX117152983 | 6.53E-05 | Sobic.010G274800 | 0.5 kb | Glycosyl transferase family 8 |
|  |  | Sobic.010G274900 | 0.5 kb | Serine/threonine-protein kinase Cx32, chloroplast precursor. |

**Table S3: Genes co-located with SNPs significantly associated with CCS (plant crop).** A distance of 0.0 kb indicates that the SNP occurs within the gene sequence. Coeff indicates the effect size on CCS units associated with the SNP. Only highly significant SNPs with closely co-located genes are listed in the table.

| **SNP**  **(* common in P and 1R)** | **P value** | **effect size** | **Sorghum orthologue** | **Distance from SNP** | **Gene family/function** |
| --- | --- | --- | --- | --- | --- |
| AX117171702 | 1.30E-04 | -0.2008 | Sobic.001G048100 | 0.0 kb | 5'-adenylylsulfate reductase-like 5. |
| AX117236275 | 1.12E-04 | -0.2594 | Sobic.003G052800 | 2.0 kb | RST1, ARM repeat superfamily protein. |
| AX117898741 | 2.27E-05 | -0.2756 | Sobic.004G303000 | 0.0 kb | O-glucosyltransferase rumi homologue. |
| AX117184164 | 1.04E-04 | -0.1918 | Sobic.006G279500 | 1.6 kb | Methyltransferase PMT15. |
| AX117970491 | 1.21E-04 | 0.1943 | Sobic.007G214700 | 0.0 kb | Fasciclin-like arabinogalactan protein 7. |
| AX117946392 | 7.44E-05 | -0.2629 | Sobic.009G024600 | 0.0 kb | Ethylene-responsive transcription factor RAP2-7. |
| AX117245638 | 4.78E-05 | 0.1627 | Sobic.009G049600 | 0.0 kb | NB-ARC domain containing protein, TOPLESS-related 2. |
| AX117245160 | 1.40E-04 | 0.1705 | Sobic.009G143400 | 0.0 kb | Scarecrow-like protein 1. |
| AX117870402 | 1.10E-04 | -0.2337 | Sobic.010G097000 | 1.6 kb | Altered xyloglucan 4. |

**Table S4: Genes co-located with SNPs significantly associated with CCS (ratoon crop).** A distance of 0.0 kb indicates that the SNP occurs within the gene sequence. Only highly significant SNPs with closely co-located genes are listed in the table.

| **SNP**  **(*common in P and 1R)** | **P value** | **Sorghum orthologue** | **Distance from SNP** | **Gene name Family/function** |
| --- | --- | --- | --- | --- |
| AX117232646 | 2.24E-05 | Sobic.002G016500 | 0.0 kb | F-box/LRR-repeat protein. |
| AX117322968 | 3.01E-05 | Sobic.002G017600 | 0.0 kb | Uncharacterised protein. |
| AX117177103 | 4.31E-05 | Sobic.003G094200 | 0.0 kb | Alanyl-tRNA synthetase. |
| AX117958788 | 9.72E-05 | Sobic.004G104100 | 0.0 kb | Ribosomal protein large subunit 16A. |
| AX117961006 | 5.44E-05 | Sobic.004G104900 | 0.0 kb | Protein kinase superfamily protein. |
| AX117309920 | 8.87E-05 | Sobic.005G084400 | 0.0 kb | La-related protein 6B, mRNA binding protein. |
| AX118054236 | 3.53E-05 | Sobic.008G018200 | 0.0 kb | Nucleotide sugar transporter-KT 1. |
| AX117938297 | 7.13E-05 | Sobic.010G215600 | 0.0 kb | Probable homogentisate phytyltransferase 1. |
